# Metabolic syndrome is associated with poor response to rifaximin in minimal hepatic encephalopathy

**DOI:** 10.1038/s41598-022-06416-z

**Published:** 2022-02-14

**Authors:** María-Pilar Ballester, Juan-José Gallego, Alessandra Fiorillo, Franc Casanova-Ferrer, Carla Giménez-Garzó, Desamparados Escudero-García, Joan Tosca, María-Pilar Ríos, Cristina Montón, Lucía Durbán, José Ballester, Salvador Benlloch, Amparo Urios, Teresa San-Miguel, Elena Kosenko, Miguel-Ángel Serra, Vicente Felipo, Carmina Montoliu

**Affiliations:** 1 Servicio de Medicina Digestiva, Hospital Clínico Universitario de Valencia, 46010 Valencia, Spain; 2 Fundación de Investigación Hospital Clínico Universitario de Valencia-INCLIVA, 46010 Valencia, Spain; 3 Laboratory of Neurobiology, Centro Investigación Príncipe Felipe, 46012 Valencia, Spain; 4 Departamento de Medicina, Universidad de Valencia, 46010 Valencia, Spain; 5 Servicio de Medicina Digestiva, Hospital Arnau de Vilanova, 46015 Valencia, Spain; 6 CIBERehd, 28029 Madrid, Spain; 7 Department of Pathology, Faculty of Medicine, University of Valencia/INCLIVA-Health Research Institute, Avda. Blasco Ibañez, 17, 46010 Valencia, Spain; 8 Institute of Theoretical and Experimental Biophysics of Russian Academy of Sciences, 142290 Pushchino, Russia

**Keywords:** Medical research, Gastroenterology, Hepatology

## Abstract

Patients with cirrhosis may show minimal hepatic encephalopathy (MHE), for which rifaximin is effective. Metabolic syndrome may be associated with cognitive impairment. Our aims were to evaluate the influence of metabolic syndrome features on response to rifaximin for neurological and inflammatory alterations in MHE. A prospective cohort study was conducted in 63 cirrhotic patients and 30 controls from two tertiary centres recruited between 2015 and 2019. Metabolic syndrome was defined according to the Adult Treatment Panel-III. Patients were classified into 31 without and 32 with MHE according to the Psychometric Hepatic Encephalopathy Score (PHES). All participants performed specific psychometric tests, and inflammatory parameters were studied. Patients with MHE received rifaximin (400 mg/8 h). Response was evaluated by PHES at 3 and 6 months. Response according to metabolic syndrome manifestations was compared. The response rate was 66%. Older age (*p* = 0.012) and all metabolic syndrome diseases (*p* < 0.05) were associated with non-response, plus an increase in risk as the number of manifestations rose (*p* < 0.001). Patients with metabolic manifestations exhibited worse processing speed (*p* = 0.011), working memory (*p* = 0.005), visual coordination (*p* = 0.013) and lower proportion of activated CD4^+^ lymphocytes (*p* = 0.039) at baseline, as well as worse concentration (*p* = 0.030), bimanual coordination (*p* = 0.004) and higher levels of intermediate monocytes (*p* = 0.026), CX3CL1 (*p* < 0.05), IL-17 (*p* = 0.022), AHR (*p* = 0.010) and IgG (*p* < 0.05) at 3 and/or 6 months of rifaximin. Patients with clinical signs of metabolic syndrome have poor response to rifaximin for MHE, with a higher proportion of neurological alterations associated with a pro-inflammatory environment.

## Introduction

Minimal hepatic encephalopathy (MHE) is present in up to 80% of patients with cirrhosis^1–4^. Although patients with MHE appear asymptomatic, subtle changes occur in attention, working memory, executive decision making, psychomotor speed and coordination^5,6^. This condition has a negative impact on quality of life, with an increased risk of falls, home and car accidents, progression to overt hepatic encephalopathy, hospitalization and death^7–12^. Given that this neuropsychiatric disorder can be reversible, therapeutic strategies are of major importance.

Several randomized placebo controlled trials have demonstrated that rifaximin improves psychometric test scores, health-related quality of life and performance in driving simulator test^13,14^. It is well-tolerated with good adherence rate and no difference in the most common adverse events compared to placebo^14^.

Several metabolic syndrome clinical manifestations such as non-alcoholic fatty liver disease (NAFLD) and diabetes mellitus (DM) are characterized by a pro-inflammatory environment and may be associated with cognitive impairment^15–19^. Additionally, metformin treatment for patients with type 2 DM seems to protect against hepatic encephalopathy^20^. Nevertheless, response to rifaximin for MHE in this high risk group of patients with metabolic syndrome features has not been well established.

We hypothesized that patients with metabolic syndrome manifestations would have a poor response to rifaximin for MHE, associated with higher levels of systemic inflammation.

The main aim of the study was to evaluate the influence of metabolic syndrome manifestations on response to rifaximin in MHE patients. Secondary goals were to analyse: (I) specific cognitive and motor alterations and (II) inflammatory parameters associated with metabolic syndrome features and their response to rifaximin treatment.

## Results

### Study population and response to rifaximin

A total of 63 patients with cirrhosis (32 of these with MHE) and 30 healthy voluntary controls were included (Table 1). Baseline PHES was 0.7 ± 0.2, − 0.8 ± 0.3 and − 7.8 ± 0.6 in controls and patients without and with MHE, respectively.

**Table 1:** Baseline characteristics of the study population.

Parameters	Controls (n = 30)	No MHE (n = 31)	MHE (n = 32)	*p* value (MHE *vs*. no MHE)
**Clinical factors**				
Age (y), mean (SEM)	59 (1)	61 (1)	62 (2)	0.499
Sex: male, n (%)	18 (60)	25 (81)	27 (84)*	0.697
**Metabolic manifestations, n (%)**				
Metabolic syndrome	3 (19)	4 (13)	6 (19)	0.508
Obesity	5 (36)	9 (53)	7 (44)	0.598
Hypertension	6 (38)	10 (32)	10 (31)	0.929
Diabetes	1 (6)	10 (32)	17 (53)**	**0.073**
Dyslipidemia	13 (81)	10 (32)**	10 (31)**	0.929
**Comorbidities, n (%)**				
Heart disease	2 (13)	3 (8)	2 (6)	1.000
Lung disease	0 (0)	5 (16)	5 (16)	1.000
Chronic kidney disease	1 (7)	0 (0)	2 (6)	–
Age at diagnosis of cirrhosis (y), mean (SEM)	–	56 (2.3)	50 (4.8)	0.327
**Etiology of cirrhosis, n (%)**	–			
Alcohol		16 (52)	17 (53)	0.904
Hepatitis C virus		13 (42)	5 (16)	**0.021**
NAFLD		1 (3)	5 (16)	0.196
Other^		1 (3)	5 (16)	0.196
**Other treatments, n (%)**	–			
Proton-pump inhibitor		13 (42)	13 (41)	1.000
Metformin		5 (16)	9 (28)	0.198
Benzodiazepine		6 (19)	5 (16)	0.785
Nonselective beta-blocker		9 (29)	7 (22)	0.613
Furosemide		4 (13)	11 (36)	**0.031**
Spironolactone		6 (19)	14 (47)	**0.023**
Norfloxacin		1 (3)	4 (13)	0.195
Lactulose		4 (13)	4 (13)	1.000
Portal hypertension, n (%)	–	27 (96)	29 (91)	0.616
**Child–Pugh, n (%)**	–			0.502
A		23 (74)	19 (59)	0.490
B		7 (23)	11 (34)	0.300
C		1 (3)	2 (6)	0.573
MELD score, mean (SEM)	–	9 (1)	10 (1)	0.509
Decompensated cirrhosis, n (%)	–	9 (29)	21 (66)	**0.002**
Portal thrombosis, n (%)	–	3 (10)	3 (9)	1.000

**^**Hepatitis B virus, primary biliary cholangitis, autoimmune, hemochromatosis or cryptogenic cirrhosis. All biochemical values are expressed as mean (SEM).*significant differences from controls (*p < 0.05; **p < 0.01; ***p < 0.001). Abbreviations: MHE and No MHE: patients with or without minimal hepatic encephalopathy, respectively; y: years; SEM: standard error of the mean; M/F: male/female; NAFLD: non-alcoholic fatty liver disease.

Significance values are [bold].

According to PHES score at 3 months, 21 (66%) of the 32 patients that received rifaximin responded to treatment. None of non-responders at 3 months improved at 6 months. Mean PHES in responders was − 7.3 ± 0.7, − 3.2 ± 0.3 and − 3.3 ± 0.6 at study initiation and at 3 and 6 months of rifaximin, respectively. Mean PHES in non-responders was − 8.7 ± 1.1, − 8.3 ± 0.9 and − 10.5 ± 0.9 at baseline, 3 and 6 months of treatment, respectively. No significant differences in baseline PHES were found between responders and non-responders (*p* = 0.281).

Mean duration of treatment was 20 ± 3 months. Adverse events were registered in four (14%) patients (two headaches, one dizziness and one aggressiveness), with drug withdrawal in three cases (two non-responders) associated with symptomatic improvement. Adherence rate was 72% and was associated with better response to therapy (78% vs. 29% in the adherent and non-adherent groups, respectively; OR = 8.8; 95CI = 1.2–63.4; *p* = 0.021).

Mean follow-up from study inclusion until loss to follow-up, death or study closure was 33 ± 3 months. Only one patient (3%) was lost at follow-up, and six (19%) died during the study period. No differences were observed on follow-up between responding and non-responding groups (log-rank = 0.870; *p* = 0.351).

### Influence of metabolic syndrome manifestations

Clinical factors according to rifaximin response are shown in Table 2. Risk factors of non-response to treatment were older age at study inclusion (mean difference 95CI = 1.7–12), all metabolic manifestations including metabolic syndrome (OR = 25; 95CI = 2.3–176), obesity (OR = 20; 95CI = 1.4–282), hypertension (OR = 8.5; 95CI = 1.5–49.5), DM (OR = 2.1; 95CI = 1.3–3.5), dyslipidemia (OR = 8.5; 95CI = 1.5–49.5) and NAFLD (OR = 14; 95CI = 1.3–150), and treatment with metformin (OR = 12; 95CI = 1.9–76.2) (Table 2). Accordingly, significantly higher overnight fasting glucose levels were observed in non-responding patients (mean difference 95CI = 12–64; *p* = 0.006) (Supplementary Table S1). The mean number of manifestations was 1 ± 0.2 in responders and 3 ± 0.4 in non-responders (mean difference 95CI = 1.3–3.2; *p* < 0.001). Moreover, a linear association was seen between number of metabolic manifestations and risk of non-response to rifaximin, so that 0%, 14%, 29%, 33% and 100% of patients with 0, 1, 2, 3 and ≥ 4 manifestations, respectively, failed to respond to treatment (*p* < 0.001). Clinical factors in patients with and without metabolic manifestations are shown in Table 2. No differences were observed in adherence depending on whether or not patients had metabolic manifestations (OR = 0.262; 95CI = 0.026–2.664; *p* = 0.362).

**Table 2:** Clinical factors according to rifaximin response and metabolic syndrome manifestations.

Clinical factor	Responders (n = 21)	Non responders (n = 11)	*p* value	Non metabolic manifestations (n = 11)	Metabolic manifestations (n = 21)	*p* value
Age (y), mean (SEM)	60 (2)	67 (2)	**0.012**	57 (2)	65 (1)	**0.003**
Sex: male, n (%)	18 (86)	9 (82)	1.000	11 (100)	16 (76)	0.138
Metabolic manifestations, n (%)	11 (52)	10 (91)	**0.029**			
Metabolic syndrome	1 (5)	5 (45)	**0.005**	–-	–-	–-
Obesity	2 (10)	5 (45)	**0.035**	–-	–-	–-
Hypertension	4 (19)	6 (55)	**0.030**	–-	–-	–-
Diabetes	8 (38)	9 (82)	**0.001**	–-	–-	–-
Dyslipidaemia	4 (19)	6 (55)	**0.030**	–-	–-	–-
**Comorbidities, n (%)**						
Heart disease	1 (5)	1 (10)	1.000	0 (0)	2 (10)	–-
Lung disease	2 (11)	3 (33)	0.295	1 (9)	4 (19)	0.636
Chronic kidney disease	0 (0)	2 (20)	–-	0 (0)	2 (10)	–-
Age at diagnosis of cirrhosis (y), mean (SEM)	50 (3)	62 (6)	0.070	45 (6)	58 (3)	0.051
**Aetiology of cirrhosis, n (%)**						
Alcohol	12 (57)	5 (46)	0.529	8 (73)	9 (43)	0.108
Hepatitis C virus	4 (19)	1 (9)	0.637	1 (9)	4 (19)	0.637
NAFLD	0 (0)	5 (46)	**0.002**	0 (0)	5 (24)	**0.078**
Other^	5 (24)	0 (0)	0.138	2 (18)	3 (14)	1.000
**Other treatments, n (%)**						
Proton-pump inhibitor	7 (33)	6 (55)	0.449	3 (27)	10 (48)	0.275
Metformin	3 (14)	6 (55)	**0.030**	0 (0)	9 (43)	**0.013**
Benzodiazepine	3 (14)	2 (18)	1.000	1 (9)	4 (19)	0.640
Nonselective beta-blocker	5 (24)	2 (18)	1.000	4 (36)	3 (14)	0.181
Diuretics	11 (52)	6 (55)	1.000	7 (63)	10 (48)	0.440
Norfloxacin	3 (14)	1 (9)	1.000	2 (18)	2 (10)	0.584
Lactulose	2 (10)	2 (18)	0.584	2 (18)	2 (10)	0.584
Portal hypertension, n (%)	18 (86)	11 (100)	0.188	11 (100)	18 (86)	0.534
**Child–Pugh, n (%)**			0.395			0.489
A	11 (52)	8 (73)		7 (64)	12 (57)	
B	9 (43)	2 (18)		4 (36)	7 (33)	
C	1 (5)	1 (9)		0 (0)	2 (10)	
MELD score, mean (SEM)	10 (1)	10 (1)	0.795	9 (1)	10 (1)	0.500
Decompensated cirrhosis, n (%)	14 (67)	7 (64)	1.000	8 (73)	13 (62)	0.703
Portal thrombosis, n (%)	2 (10)	1 (9)	1.000	1 (9)	2 (10)	1.000

**^**Hepatitis B virus, primary biliary cholangitis, autoimmune, hemochromatosis or cryptogenic cirrhosis. Abbreviations: y: years; SEM: standard error of the mean; M/F: male/female; NAFLD: non-alcoholic fatty liver disease.

Significance values are [bold].

In multivariable analysis including age (OR = 1.05; 95CI = 1.005–1.097; *p* = 0.030) and metabolic manifestations (OR = 0.045; 95CI = 0.002–0.912; *p* = 0.043), both remained as independent predictors of response to rifaximin.

Ammonia levels showed no significant differences between baseline (44 ± 8 vs. 37 ± 12; 95CI =  − 37.3–22.7; *p* = 0.620) and 3 months of rifaximin treatment (44 ± 7 vs. 40 ± 10; 95CI =  − 30–22; *p* = 0.769) but were significantly higher at 6 months (52 ± 10 vs. 24 ± 6; 95CI =  − 54–1.7; *p* = 0.038) in patients with metabolic syndrome-related disease than in those without this condition (Table 3).

**Table 3:** Biochemical parameters at baseline and follow-up according to metabolic syndrome manifestations.

Biochemical parameter	Controls (n = 30)	Patients (n = 63)		Baseline		3-month follow-up		6-month follow-up	
		No MHE (n = 31)	MHE (n = 32)	Non metabolic manifestation (n = 11)	Metabolic manifestation (n = 21)	Non metabolic manifestation (n = 11)	Metabolic manifestation (n = 21)	Non metabolic manifestation (n = 11)	Metabolic manifestation (n = 21)
Ammonia μmol/L	9.5 (1)	23 (4)**	41 (7)***^/α^	37 (12)*	44 (8)***^/α^	40 (10)*	44 (7)***^/α^	24 (7)	52 (10)***^/αα/β^
Glucose (mg/dL)	109 (5)	120 (10)	120 (9)	91 (6)*	127 (8)^β^	84 (4)**^/αα^	142 (11)**^/α/βββ^	93 (7)	157 (13)**^/α/βββ^
**Nutritional parameters**									
Cholesterol (mg/dL)	223 (11)	170 (13)**	183 (8)**	172 (14)**	188 (9)*	166 (5)***	183 (8)**	178 (8)**	186 (12)*
Triglycerides (mg/dL)	119 (12)	143 (31)	98 (8)	77 (5)**	109 (10)^ββ^	68 (10)*	125 (14)^β^	76 (8)**^/α^	134 (16)^β^
Proteins (g/dL)	7.2 (0.1)	7.3 (0.2)	7.4 (0.1)	7.4 (0.4)	7.5 (0.1)	7.6 (0.3)	7.3 (0.1)	7.6 (0.2)	6.7 (0.5)
**Kidney function**									
Urea (mg/dL)	38 (3)	37 (4)	35 (2)	30 (3)*	38 (3)	31 (5)	40 (5)	27 (3)*	35 (4)
Creatinine (mg/dL)	0.8 (0.05)	0.8 (0.05)	0.8 (0.04)	0.8 (0.04)	0.9 (0.06)	0.9 (0.1)	0.9 (0.1)	0.9 (0.1)	0.9 (0.1)
Sodium (mEq/L)	138 (0.3)	139 (0.8)	138 (0.8)	137 (1.3)	138 (1)	140 (0.5)	139 (1)	138 (1.6)	139 (1)
**Liver test**									
AST (U/L)	25 (1)	50 (6)***	40 (4)***	38 (4)**	42 (5)**	43 (5)**	43 (5)**	42 (6)*	44 (6)**
ALT (U/L)	24 (2)	34 (3)**	31 (2)*	32 (6)	31 (2)*	33 (4)*	30 (2)*	35 (6)	29 (2)
GGT (U/L)	32 (7)	109 (20)**	80 (10)***	85 (14)**	78 (14)**	95 (21)*	68 (10)**^/α^	76 (13)**	76 (12)**
ALP (mU/mL)	76 (9)	125 (12)**	132 (11)***	124 (21)	136 (14)**	138 (26)*	127 (10)**	111 (21)	135 (16)**
**Liver function**									
Bilirubin (mg/dL)	0.6 (0.02)	1.3 (0.2)**	1.7 (0.4)**	1.4 (0.4)*	1.8 (0.6)	1.2 (0.1)***	1.9 (0.6)*	1.1 (0.1)***	1.8 (0.5)*
Albumin (g/dL)	4.6 (0.07)	3.8 (0.1)***	3.7 (0.1)***	3.9 (0.2)**	3.7 (0.1)***	4 (0.2)*	3.6 (0.1)***	4 (0.2)*	3.5 (0.2)***
INR	1.0 (0.0)	1.2 (0.1)**	1.2 (0.0)***	1.2 (0.1)**	1.2 (0.05)***	1.2 (0.06)*	1.2 (0.05)***	1.2 (0.06)*	1.3 (0.07)
**Blood count**									
Leucocytes (× 10^9^/L)	6.80 (0.7)	5.69 (0.4)	5.4 (0.4)	6.2 (0.6)	4.9 (0.5)*	5.8 (0.5)	4.4 (0.3)**	6.5 (0.5)	4.9 (0.4)*^/β^
Haemoglobin (g/dL)	14.6 (0.2)	13.4 (0.4)*	13.4 (0.3)**	13.7 (0.4)	13.3 (0.5)*	13.9 (0.4)	12.9 (0.6)**	14.1 (0.5)	13.0 (0.5)*
Platelets (× 10^9^/L)	241 (15)	116 (11)***	119 (11)***	132 (26)***	111 (11)***	109 (12)***	104 (12)***	135 (19)***	107 (11)***

All values are expressed as mean (SEM). *significant differences from controls (*p < 0.05; **p < 0.01; ***p < 0.001). α significant differences from no MHE (^α^*p* < 0.05; ^αα^*p* < 0.01; ^ααα^*p* < 0.001). ^β^significant differences from patients without metabolic syndrome manifestations (^β^*p* < 0.05; ^ββ^*p* < 0.01; ^βββ^*p* < 0.001). ^∂^significant differences from baseline (^∂^*p* < 0.05; ^∂∂^*p* < 0.01; ^∂∂∂^*p* < 0.001). Abbreviations: AST: aspartate aminotransferase; ALT: alanine aminotransferase; GGT: gamma glutamyl transferase; ALP: Alkaline phosphatase; INR: international normalized ratio.

Mean follow-up from study inclusion until loss to follow-up, death or study closure showed no differences either by presence of any metabolic manifestation (log-rank = 0.732; *p* = 0.392) or by manifestation subtypes (*p* > 0.05).

### Cognitive and motor alterations

Considering specific cognitive and motor alterations, MHE patients showed worse performance in all psychometric tests compared to patients without MHE and controls. Although less marked, patients without MHE showed cognitive and motor impairment compared to controls (Table 4, and Supplementary Table S2).

**Table 4:** Psychometric test performance at baseline and follow-up according to metabolic syndrome manifestations.

Psychometric test	Controls (n = 30)	Patients (n = 63)		Baseline		3-month follow-up		6-month follow-up	
		No MHE (n = 31)	MHE (n = 32)	Non metabolic manifestation (n = 11)	Metabolic manifestation (n = 21)	Non metabolic manifestation (n = 11)	Metabolic manifestation (n = 21)	Non metabolic manifestation (n = 11)	Metabolic manifestation (n = 21)
**Cognitive test**									
**Stroop test**									
Congruent task	115 (2.6)	105 (3.2)*	71 (4)***/^ααα^	72 (9)***^/αα^	70 (4)***^/ααα^	89 (7)***^/αα^	70 (4)***^/ααα/β^	85 (7)***^/αα^	78(6)***^/ααα^
Neutral task	83 (2.4)	75 (2.3)*	56 (3)***/^ααα^	62 (6)***^/α^	53 (2)***^/ααα^	66 (4)**	55 (2)***^/ααα/β^	61 (3)***^/α^	61(4)***^/αα^
Incongruent task	46 (1.5)	41 (2)*	29 (2)***/^ααα^	31 (5)***^/α^	28 (2)***^/ααα^	34 (2)***^/α^	32 (2)***^/αα^	32 (3)***^/α^	36 (3)**
**d2 test**									
Total responses	402 (16)	337 (13)**	244 (17)***/^ααα^	276 (19)***^/α^	230 (22)***^/ααα^	317 (20)**	245 (23)***^/ααα/β^	304 (17)**/^∂^	266(8)***^/α^
Total correct	150 (6.4)	130 (6)*	83 (7)***/^ααα^	95 (6)***^/αα^	78 (10)***^/ααα^	107 (13)**	93 (10)***^/αα/∂^	103 (9)**^/α^	100(4)***^/α^
Omission errors	17 (2.6)	12 (2)	21 (4)	24 (10)	20 (5)	28 (7)	11 (2)	25 (11)	11(3)
Commission errors	1 (0.2)	4 (1)*	11 (3)**/^α^	12 (6)	10 (3)*	5 (3)	4 (2)	9 (6)	7(2)*
Total effectiveness	371 (19)	298 (16)**	214 (16)***/^αα^	241 (10)**	202 (23)***^/αα^	284 (25)*	230 (25)***^/α^	270 (14)*	149(1)**
Concentration index	146 (6.7)	126 (6)*	72 (9)***/^ααα^	83 (11)***^/αα^	68 (11)***^/ααα^	102 (14)**	89 (11)***^/α/∂^	94 (14)**^/α^	94 (15)**
Oral SDMT	50 (1.3)	44 (2)**	23 (2)***/^ααα^	32 (2)***^/ααα^	19 (3)***^/ααα/ββ^	36 (2)***^/α^	24 (3)***^/ααα/ββ^	34 (3)***^/α^	31 (4)***^/αα^
Digit Span	16 (0.8)	13 (0.5)**	10 (0.5)***/^αα^	12 (1)**	10 (1)***^/ααα/β^	13 (1)	10 (1)***^/ααα/β^	14 (2)	11 (1)**
Letter-number sequencing	10 (0.4)	8 (0.5)**	5 (0.5)***/^ααα^	7 (1)**	4 (1)***^/ααα/ββ^	7 (1)**	6 (1)***^/αα^	6 (1)**	5 (1)***^/αα^
**Motor coordination tests**									
Bimanual coordination	1.9 (0.03)	2.3 (0.1)***	3.7 (0.4)***/^αα^	2.9 (0.2)**^/αα^	3.9 (0.5)***^/αα^	2.6 (0.1)***^/α^	3.3 (0.2)***^/ααα/ββ^	2.7 (0.1)**^/α^	3.3 (0.3)***^/αα^
Visuo-motor coordination	2.4 (0.1)	3 (0.1)***	3.9 (0.2)***/^ααα^	3 (0.2)***	4 (0.2)***^/ααα/β^	3.4 (0.3)***	4.1 (0.4)***^/αα^	3.4 (0.2)***	3.9 (0.4)***^/α^

All values are expressed as mean (SEM). *significant differences from controls (*p < 0.05; **p < 0.01; ***p < 0.001). α significant differences from no MHE (^α^*p* < 0.05; ^αα^*p* < 0.01; ^ααα^*p* < 0.001). ^β^significant differences from patients without metabolic syndrome manifestations (^β^*p* < 0.05; ^ββ^*p* < 0.01; ^βββ^*p* < 0.001). ^∂^significant differences from baseline (^∂^*p* < 0.05; ^∂∂^*p* < 0.01; ^∂∂∂^*p* < 0.001). Abbreviations: MHE and No MHE: patients with or without minimal hepatic encephalopathy, respectively.

**Figure 1:**
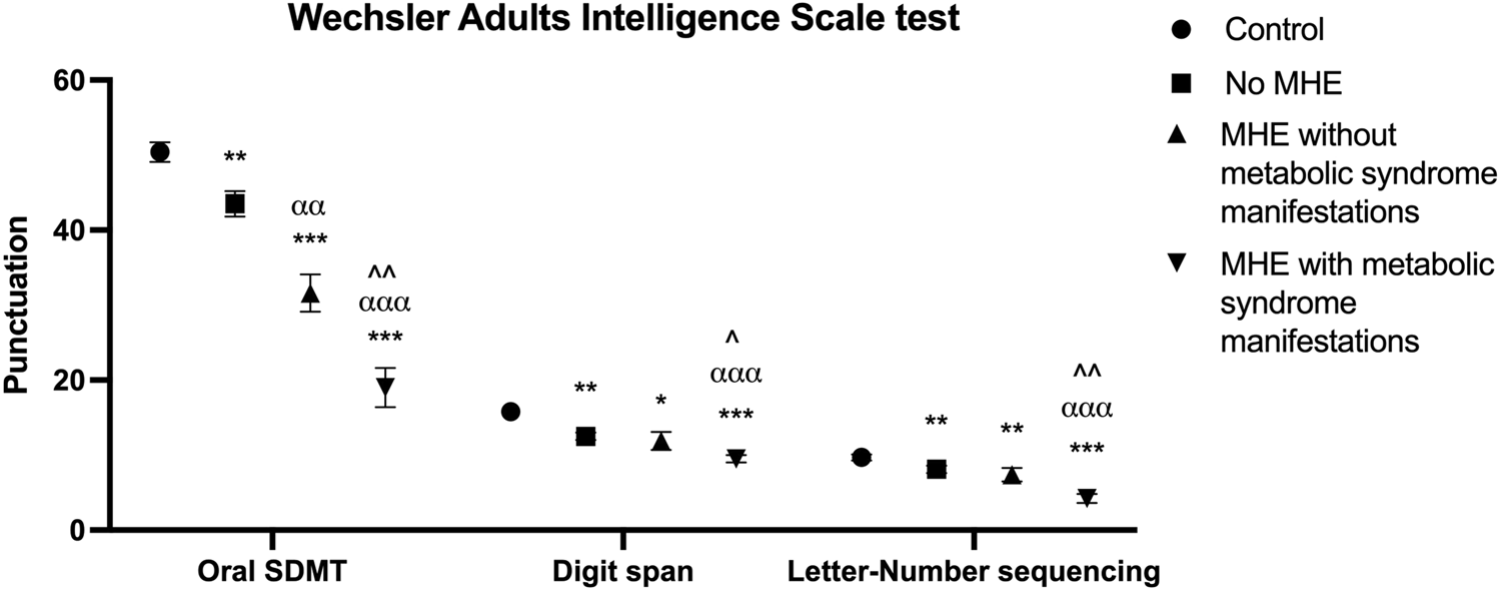
Performance of specific oral psychometric tests by controls, patients without MHE and patients with MHE according to presence of manifestations of metabolic syndrome at baseline. Oral SDMT: oral version of Symbol digit modalities test, expressed in correct pairings. Digit Span and Letter-Number sequencing test are expressed as right answers. Punctuation expressed as mean (SEM). *significant differences from controls (*p < 0.05; **p < 0.01; ***p < 0.001). ^α^significant differences from no MHE (^α^*p* < 0.05; ^αα^*p* < 0.01; ^ααα^*p* < 0.001). ^significant differences from patients with MHE without metabolic syndrome manifestations (^*p* < 0.05; ^^*p* < 0.01; ^^^*p* < 0.001).

Among patients with MHE, those with or without signs of metabolic syndrome did not show significant differences in baseline PHES (− 8.2 ± 0.7 vs. − 7.3 ± 1.1; *p* = 0.510) but displayed worse mental processing speed and selective attention in Oral SDMT (95CI = 4–22; *p* = 0.008); working memory with Digit Span (95CI = 0.2–4.6; *p* = 0.037); letter-number sequencing test (95CI = 1.1–5.4; *p* = 0.005) (Fig. 1), and visuo-motor coordination (95CI = 0.2–1.3; *p* = 0.013) (Table 4). Moreover, there was a significant correlation between increasing number of metabolic signs and worse performance in these tests: in Oral SDMT (r =  − 0.438; *p* = 0.015), Digit Span (r =  − 0.401; *p* = 0.028), letter-number sequencing (r =  − 0.404; *p* = 0.033) and visuo-motor coordination (r = 0.431; *p* = 0*.*025) at baseline.

**Figure 2:**
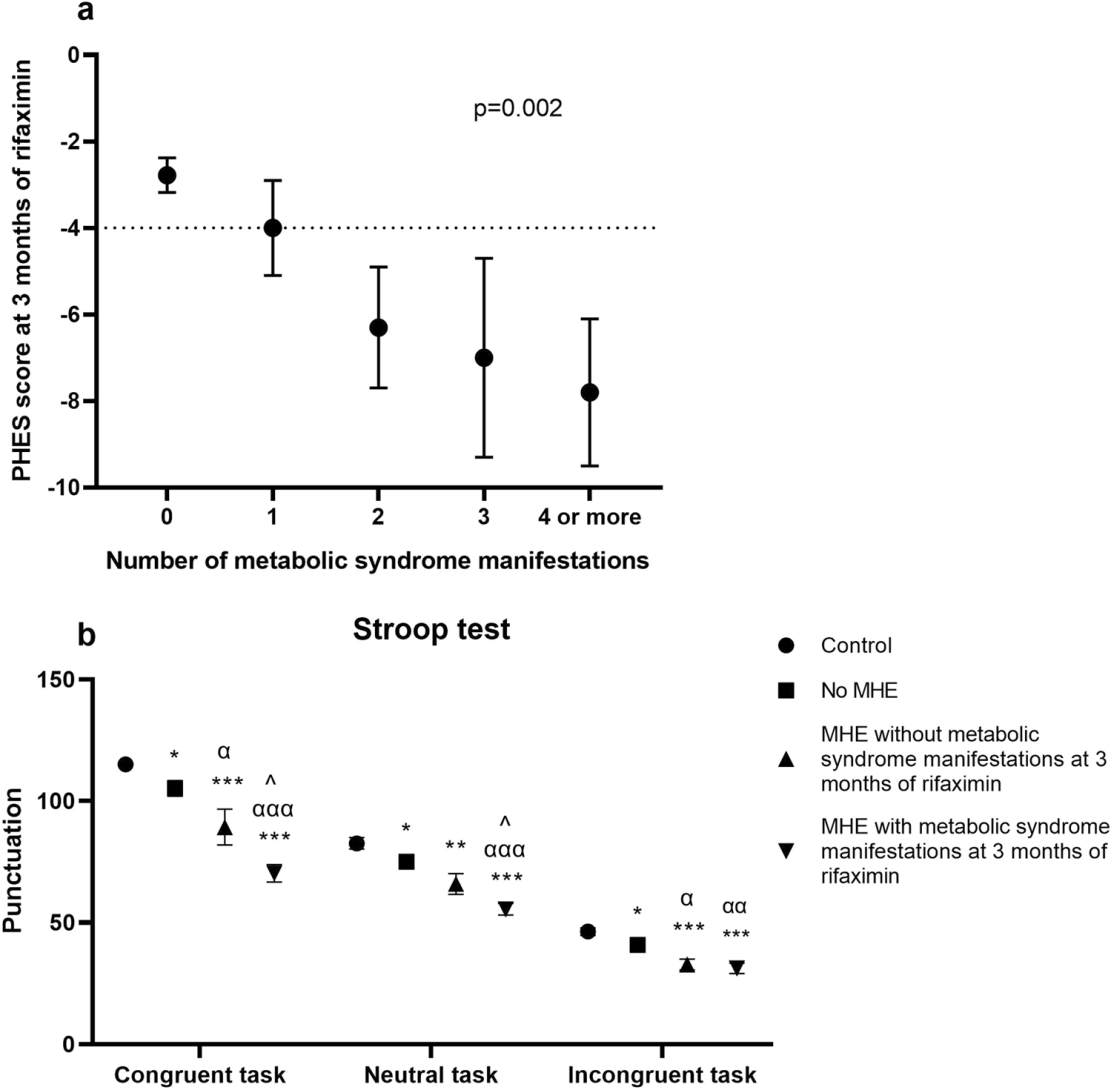
(**a**) Correlation between PHES and categorized number of metabolic manifestations at 3 months of rifaximin treatment. (**b**) Performance of Stroop test by controls, patients without MHE and patients with MHE according to presence of manifestations of metabolic syndrome at 3 months of rifaximin treatment. Stroop test: Congruent task: number of words read in 45 s; Neutral task: number of colours read in 45 s; Incongruent task: number of items completed in 45 s. Punctuation expressed as mean (SEM). *significant differences from controls (*p < 0.05; **p < 0.01; ***p < 0.001). α significant differences from no MHE (^α^*p* < 0.05; ^αα^*p* < 0.01; ^ααα^*p* < 0.001). ^significant differences from patients with MHE without metabolic syndrome manifestations (^*p* < 0.05; ^^*p* < 0.01; ^^^*p* < 0.001).

At 3-month of rifaximin treatment, patients without manifestations of metabolic syndrome produced a significantly better PHES score than patients with manifestations (− 2.78 ± 0.4 vs. − 5.9 ± 0.7; 95CI = 1.4–4.8; *p* = 0.001). A strong inverse correlation was observed between PHES and number of metabolic syndrome-related alterations (r =  − 0.623; *p* < 0.001) with reduced mean PHES score across categorized numbers of metabolic signs (− 4 ± 1.1, − 6.3 ± 1.4, − 7 ± 4 and − 7.6 ± 1.3 in patients with 1, 2, 3 and ≥ 4 manifestations, respectively; *p* = 0.002) (Fig. 2a). Patients with metabolic syndrome-related alterations not only failed to improve in psychometric test scores at 3 months of rifaximin treatment, but also showed slower mental processing speed and selective attention, with worse performance in the total number of words (95CI = 4–34; *p* = 0.015) and colours (95CI = 0.9–20; *p* = 0.032) in the Stroop test (Fig. 2b), worse concentration with total responses (95CI = 7.5–136; *p* = 0.030) in the d2 test, and worse bimanual coordination (95CI = 0.2–1.2; *p* = 0.007) (Table 4). Patients performed progressively worse in these tests as the number of metabolic manifestations increased: in total number of words (r =  − 0.395; *p* = 0.038) and colours (r =  − 0.375; *p* = 0.049) in the Stroop test, total responses (r =  − 0.467; *p* = 0.019) in the d2 and in bimanual coordination test (r = 0.528; *p* = 0.004).

At 6-month follow-up, patients with signs of metabolic syndrome showed a trend towards lower PHES compared to patients without these manifestations (− 6.3 ± 1.2 vs. − 3.6 ± 0.6; 95CI =  − 0.2–5.6; *p* = 0.066). A moderate inverse correlation was maintained between PHES and number of metabolic syndrome manifestations (r =  − 0.465; *p* = 0.034).

### Inflammatory parameters

Treatment with rifaximin reduced the percentage of intermediate proinflammatory monocytes (CD14^++^CD16^+^), autoreactive T CD4^+^ lymphocytes (CD4^+^CD28^−^) and increased classical monocytes (CD14^++^CD16^−^) and non-autoreactive T CD4^+^ lymphocytes (CD4^+^CD28^+^) to almost normal values at 6 months in the responding group. Regarding percentage of activated T CD4^+^ lymphocytes (CD69), responding patients presented elevated baseline levels which were reversed with rifaximin, in contrast with the absence of elevated activated levels at study start or during follow-up in non-responding patients. Levels of IgG at the beginning of treatment were similar in both groups of response, but these levels dropped at 3 and 6 months in responding patients, with significant differences compared to non-responding patients (Supplementary Table S3).

**Figure 3:**
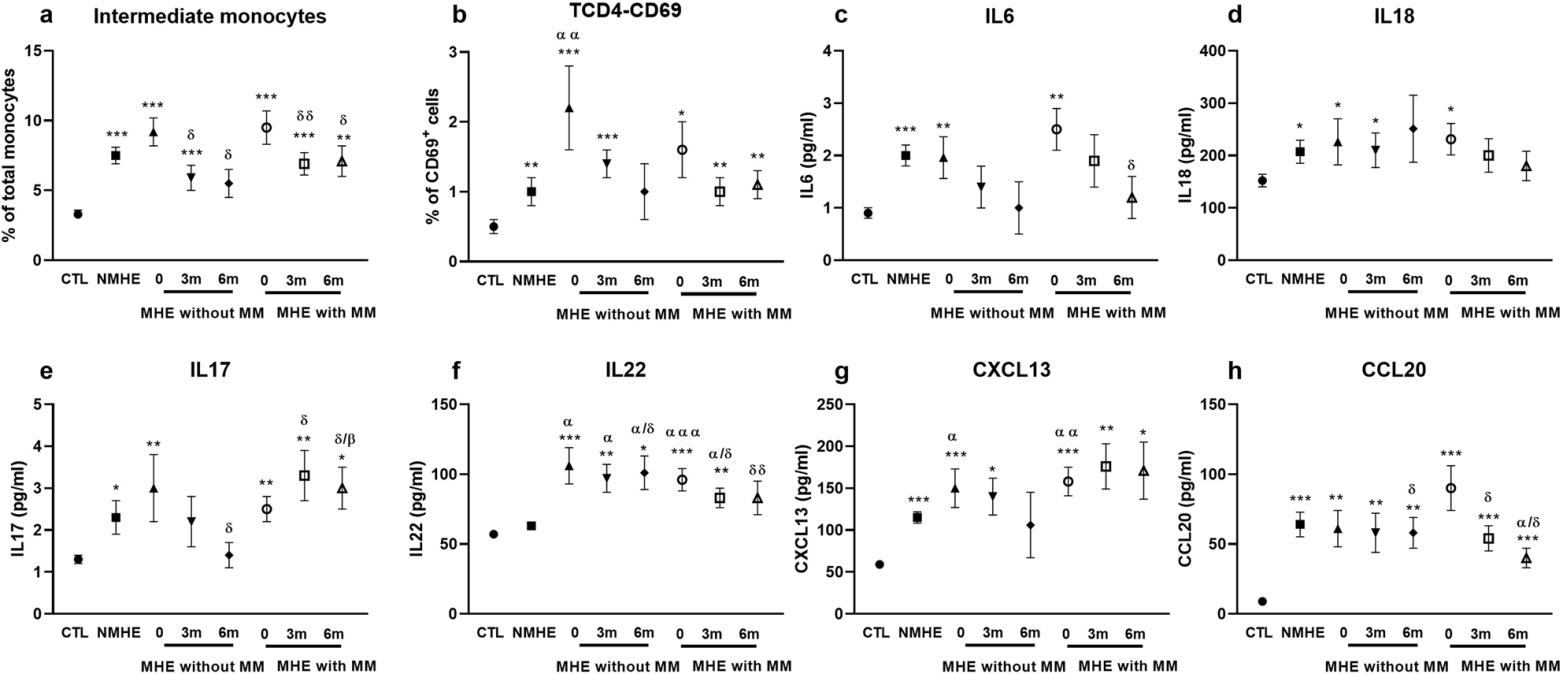
Inflammatory parameters at baseline and at 3 and 6 months of rifaximin treatment according to metabolic syndrome manifestations. *significant differences from controls (*p < 0.05; **p < 0.01; ***p < 0.001). ^α^significant differences from no MHE (^α^*p* < 0.05; ^αα^*p* < 0.01; ^ααα^*p* < 0.001). ^β^Significant differences from patients without metabolic syndrome manifestations (^β^*p* < 0.05; ^ββ^*p* < 0.01; ^βββ^*p* < 0.001). ^∂^significant differences from baseline (^∂^*p* < 0.05; ^∂∂^*p* < 0.01; ^∂∂∂^*p* < 0.001). Abbreviations: CTL: controls; MHE and NMHE: patients with or without minimal hepatic encephalopathy, respectively; MM: metabolic syndrome manifestations.

Inflammatory parameters in patients with and without metabolic syndrome manifestations are described in Table 5 and Fig. 3. Rifaximin treatment reduced serum levels of several proinflammatory interleukins (IL) such as IL22 and CCL20 in both groups of patients, while IL17 and CXCL13 were only reduced in patients without metabolic syndrome manifestations.

**Table 5:** Inflammatory parameters at baseline and follow-up according to metabolic syndrome manifestations.

Biochemical parameters	Controls (n = 30)	Patients (n = 63)		Baseline		3-month follow-up		6-month follow-up	
		No MHE (n = 31)	MHE (n = 32)	Non metabolic manifestation (n = 11)	Metabolic manifestation (n = 21)	Non metabolic manifestation (n = 11)	Metabolic manifestation (n = 21)	Non metabolic manifestation (n = 11)	Metabolic manifestation (n = 21)
**Immunophenotype study**									
**Monocytes**^**a**^									
Classical	92.2 (0.7)	88.7 (0.8)**	89.7 (0.9)*	91 (1.6)	89 (1.2)*	91 (1.1)	91 (1.2)	92 (1.1)^α^	92 (1.1)^α/∂^
Intermediate	3.3 (0.3)	7.5 (0.6)***	9.4 (0.8)***/^α^	9.2 (1.0)***	9.5 (1.2)***	5.9 (0.9)***^/∂^	6.9 (0.8)***^/∂∂^	5.5 (1.0)^∂^	7.1 (1.1)**^/∂^
Non-classical	2 (0.3)	2.1 (0.3)	2.7 (0.4)	2.4 (0.6)	2.8 (0.6)	1.7 (0.5)	0.7(0.2)***^/ααα /β/∂∂∂^	0.5 (0.3)**^/ααα^	0.6 (0.2)***^/ ααα/∂∂^
**CD4**^**+**^**T lymphocytes**									
Autoreactive^b^	8 (1)	10 (1)	12 (2)*	9.9 (3)*	13 (2)*	12 (2)*	15 (4)	7 (1)	9 (3)^∂^
Non-autoreactive^b^	92 (3)	90 (1)	88 (2)	90 (3)	87 (2)	88 (5)	84 (5)	91 (3)	91 (3)
Activated^c^	0.5 (0.1)	1 (0.2)**	2 (0.4)**/^α^	2.2 (0.6)***/ ^α α^	1.6 (0.4)*	1.4 (0.2)***	1.0 (0.2)**	1 (0.4)	1.1 (0.2)**
**Cytokines**^**d**^									
IL-6	0.9 (0.1)	2.0 (0.2)***	2.2 (0.3)***	1.9 (0.4)**	2.5 (0.4)**	1.4 (0.4)	1.9 (0.5)	1.0 (0.5)	1.2 (0.4)^∂^
IL-18	152 (12)	207 (22)*	229 (25)**	226 (44)*	231 (30)*	210 (33)*	200 (32)	251 (64)	180 (28)
IL-15	2.9 (0.1)	3.3 (0.1)*	3.4 (0.1)*	3.6 (0.2)*	3.3 (0.2)	3.2 (0.2)	3.2 (0.1)	3.0 (0.2)^∂^	3.0 (0.1)^α^
IL-17	1.3 (0.1)	2.3 (0.4)*	2.7 (0.4)**	3.0 (0.8)**	2.5 (0.3)**	2.2 (0.6)	3.3 (0.6)**^/∂^	1.4 (0.3)^∂^	3 (0.5)*^/β/∂^
IL-21	155 (14)	237 (33)*	439 (143)	673 (422)	343 (109)	511 (356)	278 (79)	487 (416)	370 (119)
IL-22	57 (2.5)	63 (2.6)	99 (6.8)***/^ααα^	106 (13)**^/α^	96 (8)***^/ααα^	97 (10)**^/α^	83 (7)**^/α/∂^	101 (12)*^/α/∂^	83 (12)^∂∂^
CXCL13	59 (2.0)	115 (6.7)***	155 (13)***/^αα^	150 (23)***^/α^	158 (17)***^/αα^	140 (22)*	176 (27)**	106 (39)	171 (34)*
CX3CL1	0.6 (0.03)	0.7 (0.04)**	0.9 (0.1)***/^αα^	0.8 (0.1)**	0.9 (0.1)***^/αα^	0.8 (0.1)*	1.0 (0.1)***^/ααα^	0.8 (0.1)*	1.0 (0.1)**^/α/∂^
CCL20	8.9 (0.9)	64 (8.8)***	79 (11)***	61 (13)**	90 (16)***	58 (14)**	54 (9)***^/∂^	58 (11)**^/∂^	40 (7)***^/α/∂^
**Transcription factors**^**e**^									
BCL6	1.0 (0.1)	0.8 (0.1)*	0.9 (0.1)	0.9 (0.2)	0.9 (0.1)	0.6 (0.2)*	1.0 (0.1)	1.1 (0.2)	1.0 (0.1)
AHR	0.9 (0.1)	1.0 (0.1)	1.4 (0.2)**/^α^	1.5 (0.3)	1.4 (0.2)*	1.1 (0.2)	1.3 (0.2)**	0.9 (0.1)	0.9 (0.1)
TBX21	1.1 (0.1)	1.4 (0.1)*	1.4 (0.1)*	1.5 (0.2)	1.4 (0.1)	1.8 (0.2)*	1.3 (0.1)	1.3 (0.2)	1.3 (0.2)
GATA3	1.1 (0.1)	1.0 (0.1)	1.0 (0.1)	1.1 (0.1)	0.9 (0.1)	1.3 (0.2)	1.1 (0.1)	1.2 (0.2)	1.1 (0.2)
RORC	1.0 (0.1)	0.9 (0.1)	0.8 (0.1)	1.0 (0.3)	0.7 (0.1)*	0.9 (0.1)	0.6 (0.1)***^/α/β/∂^	0.6 (0.1)*	0.4 (0.1)***^/ ααα/β/∂^
**IgG levels**^**f**^	99 (2)	89 (20)	155 (12)**	157 (5)	153 (2)	134 (9)	142 (11)	103 (11)	141 (21)

^a^Expressed as percentage of the three subsets of monocytes over total monocyte cells. ^b^Expressed as percentage of total CD4^+^T lymphocytes. ^c^Percentage of CD4^+^ T lymphocytes that express the early activation marker CD69. ^d^Cytokine levels are expressed in pg/mL except for CX3CL1 which is in ng/mL. ^e^Data represent the normalized target gene (HPRT) amount relative to controls which are considered as 1. ^f^Percentage of variation compared to controls. All values are expressed as mean (SEM). *significant differences from controls (*p < 0.05; **p < 0.01; ***p < 0.001). α significant differences from no MHE (^α^*p* < 0.05; ^αα^*p* < 0.01; ^ααα^*p* < 0.001). ^β^Significant differences from patients without metabolic syndrome manifestations (^β^*p* < 0.05; ^ββ^*p* < 0.01; ^βββ^*p* < 0.001). ^∂^significant differences from baseline (^∂^*p* < 0.05; ^∂∂^*p* < 0.01; ^∂∂∂^*p* < 0.001). Abbreviations: MHE and No MHE: patients with or without minimal hepatic encephalopathy, respectively.

Analysing by manifestations, lower levels of baseline activated T CD4^+^ lymphocytes (CD69) were observed in patients with obesity (1 ± 0.2 vs. 2.4 ± 0.7%; *p* = 0.076) and metabolic syndrome (1 ± 0.2 vs. 2.2 ± 0.5%; *p* = 0.039).

At 3 months of treatment, CX3CL1 levels were higher in patients with hypertension (1.1 ± 0.1 vs. 0.9 ± 0.1 ng/mL; *p* = 0.070) or metabolic syndrome (1.1 ± 0.1 vs. 0.9 ± 0.1 ng/mL; *p* = 0.047). AHR expression was also higher in patients with hypertension (1.7 ± 0.2 vs. 1.0 ± 0.1; *p* = 0.010), with increased levels compared to those prior to treatment initiation. IgG levels showed a tendency to be higher in patients with hypertension (157 ± 8 vs. 127 ± 11; *p* = 0.081) or metabolic syndrome (161 ± 11 vs. 130 ± 10; *p* = 0.090), correlating positively with the increasing number of metabolic syndrome manifestations (r = 0.635; *p* = 0.066).

Six months of treatment with rifaximin induced a higher reversion rate of classical (92.6 ± 0.7 vs. 88.4 ± 2.7; *p* = 0.047) and intermediate (5.8 ± 0.8 vs. 10.7 ± 1.9; *p* = 0.026) monocytes in patients without, than with, metabolic syndrome. We also observed increased levels of IL17 in patients with metabolic manifestations (3.0 ± 0.5 vs. 1.4 ± 0.3 pg/mL; *p* = 0.022) (Fig. 3e), and of CX3CL1 in patients with dyslipidaemia (1.2 ± 0.2 vs. 0.8 ± 0.1; *p* = 0.009), hypertension (1.2 ± 0.1 vs. 0.8 ± 0.1; *p* = 0.028) or metabolic syndrome (1.3 ± 0.2 vs. 0.9 ± 0.1; *p* = 0.020). Like the results at 3 months, at 6 months of rifaximin IgG levels tended to be raised in patients with hypertension (176 ± 34 vs. 112 ± 12; *p* = 0.062), metabolic syndrome (176 ± 34 vs. 112 ± 12; *p* = 0.062) or obesity (149 ± 7 vs. 99 ± 8; *p* = 0.040).

## Discussion

The current post-approval, prospective follow-up study addresses the influence of metabolic syndrome clinical features on response to rifaximin in patients with MHE. Our findings demonstrate that metabolic syndrome-related diseases are associated with poorer response to rifaximin for management of MHE, with more cognitive and motor alterations and higher levels of inflammation compared to patients without these conditions.

Risk factors for MHE in our study were use of diuretics and decompensated cirrhosis, as previously described^21,22^, suggesting that our population was appropriate to evaluate response to rifaximin.

Rifaximin is an oral non-systemic broad spectrum antibiotic which inhibits bacterial RNA/protein synthesis by binding to bacterial DNA-dependent RNA-polymerase. Over the last decade, experimental and clinical evidence has suggested that rifaximin could modulate gut microbiota, reducing intestinal ammonia and toxin formation, and thus systemic inflammation^23–25^. Although hyperammonaemia and inflammatory response are the main pathogenic mechanisms of MHE^26^, there is still a subset of patients who do not respond to rifaximin, whose features are not well established.

The results of our study show a 66% response rate to rifaximin with a good adherence that lifted the response rate up to 78%, similar to previous clinical trials^13,14^. Response to treatment was evaluated at 3 and 6 months, with no response observed at 6 months in patients without response at 3 months indicating that no benefit is obtained by prolonging rifaximin in patients without response at 3 months.

An improvement in neurological alterations and quality of life has been reported with administration of rifaximin in patients with MHE^13,14,23^. A similar, progressive improvement of cognitive and motor alterations was observed in our treatment responder group. Nevertheless, working memory showed a subtler response, therefore, MHE patients are likely to maintain certain limitations in information storage and processing despite treatment.

Turning to rifaximin mechanisms of action, a reduction in ammonia levels occurred only non-significantly in the responding patient group, suggesting that rifaximin exerts its effect primarily by modulating the inflammatory changes associated with this entity, as has been proposed by Mangas-Losada et al.^27^. Parameters that improved selectively in responding patients were percentages of classical and intermediate monocytes, levels of IL-17, IL-21, IL-22, CXCL13 and IgG, so that reversing these alterations may be necessary to obtain a therapeutic response.

Interestingly, when analysing response-related immune alterations, the non-responding group did not present an increase in percentage of activated T CD4^+^ lymphocytes at treatment initiation. Since CD69 is an early activation marker^28,29^, it is conceivable that non-responding patients could be in a more advanced activation phase in which CD69 is not expressed, and which cannot be reversed by rifaximin. Thus, CD69 could be used as a biomarker in patient selection for treatment.

Several comorbidities, such as DM and insulin resistance, contribute to the development of OHE^18^. In other metabolic syndrome-related conditions such as NAFLD, cognitive dysfunction is an associated extrahepatic manifestation in up to 70% of cases^15–17,30,31^. The results of our study confirmed that patients with metabolic syndrome-associated disease exhibit cognitive impairment, with greater severity of these alterations as the number of manifestations increases. In response to the question of whether cognitive impairment in this group of patients was due to MHE or secondary to metabolic disorders, we could see that the control group had a similar proportion of metabolic syndrome and, nevertheless, had no cognitive abnormalities. Thus, there must be a synergy between chronic liver disease and metabolic disorders that conditions neurological alterations.

As a novel finding, patients with metabolic syndrome-related disease present poorer response to rifaximin prescribed for MHE. Also noteworthy was the fact that risk of non-response increases with the number of metabolic syndrome manifestations. Therefore, obtaining better response to rifaximin probably requires treating metabolic alterations concomitantly in this group of patients.

Several features of metabolic syndrome have been described in the pathogenesis of cognitive disturbances, such as systemic inflammation and vascular dysfunction, as well as NAFLD-linked features such as gut microbiota disruption or impairment of urea synthesis in the liver^16^. Nevertheless, the mechanisms underlying the worse treatment response in this patient subgroup are unknown. The results in our cohort showed that patients with metabolic syndrome clinical features have a more exacerbated pro-inflammatory environment that besides predisposing to neurological alterations leads to worse response to treatment. When comparing inflammatory alterations observed in patients with clinical manifestations of the metabolic syndrome to those that selectively improved in responding patients, the parameters determining lack of treatment response in this patient group may be percentage of intermediate monocytes, IL-17 and IgG levels. Intermediate (CD14^++^CD16^+^) monocytes are both producers of TNFα, IL-1β, IL-6, IL-18 and CCL20 and macrophage precursors, also with an inflammatory phenotype^32,33^. In turn monocytes and macrophages act as presenting antigen cells contributing to lymphocyte activation^34^. The humoral response plays an important role in development and progression of autoimmune and inflammatory disease^35^ and probably in MHE, demonstrated by increased IgG levels as a reflection of B lymphocyte activation in MHE patients. Moreover, patients with metabolic syndrome associated lower levels of activated T CD4^+^ lymphocytes at treatment initiation which could contribute to worse rifaximin response.

Among other factors, older age was also associated with non-response to treatment and multivariable analyses confirmed that both age and metabolic syndrome manifestations were independent predictors of response to rifaximin. For this reason, we believe that early establishment of treatment is crucial to bring benefits. Unlike in the Ampuero J study^20^, in ours, treatment with metformin was associated with higher risk of treatment non-response. Two factors could account for this: metformin treatment was prescribed in diabetic patients, and clinical data was collected transversally at the beginning of the study, a limitation that precluded establishing a causal relationship between treatment initiation and development of cognitive alterations.

The study also has several strengths to be highlighted. The findings are based on a well-characterized patient cohort, whose response to treatment was prospectively evaluated. Moreover, the study included a group of voluntary healthy controls as a reference for evaluation of neurological and inflammatory parameters. Nevertheless, further studies with blinded administration of rifaximin in patients with and without metabolic syndrome and larger sample size are warranted to confirm and establish more robust results.

In conclusion, patients with clinical signs of metabolic syndrome have poor response to rifaximin for MHE. They exhibit a higher proportion of neurological alterations which increase in severity as the number of metabolic syndrome disorders rises. This is associated with non-elevated levels of activated T CD4^+^ lymphocytes at treatment initiation and a pro-inflammatory environment not completely reversible with treatment. Careful and early evaluation, predominantly in patients with risk factors, and treating both MHE and metabolic syndrome manifestations, are crucial to obtain improvements in cognitive dysfunction in patients with cirrhosis.

## Materials and methods

### Study design and patient selection

A multicentre, post-approval study with prospective follow-up was performed. Patients with cirrhosis treated in the Hepatology Unit of two tertiary centres (Clinic University Hospital of Valencia or Arnau de Vilanova, Spain) were screened for eligibility. Diagnosis of cirrhosis was based on liver histology or a combination of clinical, biochemical and imaging signs. Exclusion criteria were previous overt hepatic encephalopathy (OHE), neurological or psychiatric disorder, alcohol or drug consumption during the last 6 months, infection or antibiotic use (other than quinolones for spontaneous bacterial peritonitis) or gastrointestinal bleeding during the last 6 weeks, active antiviral treatment, transjugular intrahepatic portosystemic shunt or hepatocellular carcinoma. Patients were recruited consecutively between 2015 and 2019 and classified as with or without MHE using the Psychometric Hepatic Encephalopathy Score (PHES)^36,37^. A random group of these patients was selected to achieve the minimum sample size. A group of healthy voluntary controls without liver disease who met the same applicable exclusion criteria were included during the same study period. Patients and controls underwent clinical evaluation, psychometric tests and blood analyses to determine ammonia levels and biochemical measurements on the same day.

### Definitions

Metabolic syndrome was defined according to the National Cholesterol Education Program Adult Treatment Panel III criteria^38^. Clinical manifestations of metabolic syndrome considered were obesity (waist circumference > 102 cm in men or > 88 cm in women); dyslipidemia (fasting triglycerides level ≥ 150 mg/dl, fasting high-density lipoprotein cholesterol level < 40 mg/dl in men or < 50 mg/dl in women, or on lipid-lowering treatment); hypertension (blood pressure ≥ 130/85 mmHg or on antihypertensive treatment); fasting blood sugar ≥ 110 mg/dl or on antidiabetic treatment, and NAFLD.

Model for End stage Liver Disease (MELD)^39^ and Child-Pugh^40^ score were used to evaluate disease severity. Cirrhosis decompensations assessment was made using clinical guideline diagnostic criteria^41^.

### Psychometric tests

Diagnosis of MHE was made with a PHES score ≤  − 4^36,37^.

Other psychometric tests performed to study specific cognitive and motor alterations were: (I) Stroop and oral version of Symbol Digit modalities test (Oral SDMT) from the Wechsler Adults Intelligence Scale to evaluate processing speed and selective attention; (II) d2 test to assess concentration; (III) Digit Span and Letter-Number sequencing tests from the Wechsler Adults Intelligence Scale to evaluate working memory; and (IV) bimanual and visuo-motor coordination tests to assess coordination^42–44^.

### Laboratory measurements

Blood ammonia was measured with the Ammonia Test Kit II for the Pocket Chem BA system (Arkray, Inc., Kyoto, Japan).

Several inflammatory parameters were studied related to immunophenotype, cytokine levels in serum, transcription factors and IgG levels in plasma by flow cytometry, ELISA, quantitative PCR and Western Blot, respectively as described by Mangas-Losada et al.^23^.

### Rifaximin treatment and follow-up

All MHE patients received treatment with rifaximin (400 mg/8 h orally) for at least 3 months. PHES was repeated at 3 months to evaluate response to treatment. Response to therapy was considered when PHES score was >  − 4. Responding patients were maintained on treatment, while in non-responders treatment was maintained or withdrawn according to patient and physician preferences. Adverse events and withdrawal due to side effects were registered. Adherence was evaluated by direct patient interview and by the electronic medical prescription system^45^. Besides PHES, other psychometric tests and biochemical measurements were repeated at 3 months in all patients and at 6 months in those who continued on treatment.

Clinical surveillance was performed at 1, 3 and 6 months after inclusion and then every 6 months thereafter unless clinical deterioration occurred, until loss to follow-up, death or study closure in January 2020.

### Statistical analysis

Differences according to rifaximin response were compared using Student’s t or Mann–Whitney U tests for continuous data and the chi-square, Fisher test or linear association for categorical data, as required. Measures of association between qualitative variables were reported as odds ratio (OR) with 95% confidence intervals (95CI) and *p* values. To study the independent contribution of each factor to rifaximin response, multivariate logistic regression analysis was performed including significant and relevant variables from the univariable analysis. Between-group comparison before and after rifaximin treatment was made using a paired t-test or Wilcoxon test for continuous data, and the McNemar test for categorical data. Follow-up was calculated with the Kaplan–Meier curve from day of study inclusion to date of censoring (loss of follow-up, death or study closure, whichever came first). Survival curves were compared by log-rank test. Correlations between number of metabolic syndrome signs and performance in psychometric tests or inflammatory levels were calculated by Spearman’s rank correlation. An ANOVA post-hoc Bonferroni test was also used to differentiate psychometric test performance and inflammatory parameters across categorized groups of metabolic manifestations. For sample size calculation, accepting an alpha risk of 0.05 and a beta risk of 0.2 in a two sided test, 22 and 11 subjects with and without metabolic syndrome manifestations were necessary to find a statistically significant proportion difference, expected to be of 0.9 in group 1 and 0.4 in group 2 with an anticipated drop-out rate of 10%. To our knowledge there are no studies that evaluate the influence of metabolic syndrome manifestations on response to Rifaximin. Therefore, parameters for proportion difference were estimated according to our past clinical experience^23^. The variable used for the calculation was the expected percentage of response in each of the study groups (with and without manifestations of the metabolic syndrome).

All tests were two sided and a *p* value < 0.05 was considered statistically significant. Analyses were performed using SPSS Statistics Version 22.

### Ethics

The study was approved by the Clinic University Hospital of Valencia Institutional Review Board in 2015 (F-CE-GEva-15) and classified by the Spanish Agency of Medicines and Medical Devices (CMF-NRT-2017). The study protocol conforms to the ethical guidelines of the 1975 Declaration of Helsinki. All participants were enrolled after signing written informed consent.

## Supplementary Information

Supplementary Information.

## Data Availability

All data generated or analysed during this study is included in the published article.
