# Supplementary material for: Metabolic syndrome is associated with poor response to rifaximin in minimal hepatic encephalopathy
Source: Sci Rep. 2022 Feb 14;12:2463. doi: 10.1038/s41598-022-06416-z (PMC8844048; doi:10.1038/s41598-022-06416-z)
Supplement: Supplementary file 1 — Supplementary Information. [file 41598_2022_6416_MOESM1_ESM.pdf]

# SUPPLEMENTARY INFORMATION

## Metabolic syndrome is associated with poor response to rifaximin in minimal hepatic encephalopathy

María-Pilar Ballester, Juan-José Gallego, Alessandra Fiorillo, Franc Casanova-Ferrer, Carla Giménez-Garzó, Desamparados Escudero-García, Joan Tosca, María-Pilar Ríos, Cristina Montón, Lucía Durbán, José Ballester, Salvador Benlloch, Amparo Urios, Teresa San-Miguel, Elena Kosenko, Miguel-Ángel Serra, Vicente Felipo, Carmina Montoliu*

Contents:

**Supplementary Table S1.** Biochemical parameters at baseline and according to rifaximin response.

**Supplementary Table S2.** Psychometric test performance at baseline and according to rifaximin response.

**Supplementary Table S3.** Inflammatory parameters at baseline and according to rifaximin response.

| Biochemical parameter | Controls (n=30) | Patients (n=63) | | Baseline | | 3-month follow-up | | 6-month follow-up | |
|---|---|---|---|---|---|---|---|---|---|
| | | No MHE (n=31) | MHE (n=32) | Responders (n=21) | Non responders (n=11) | Responders (n=21) | Non responders (n=11) | Responders (n=19) | Non responders (n=9) |
| **Ammonia μmol/L** | 9.5 (1) | 23 (4)$^{**}$ | 41 (7)$^{***/\alpha}$ | 42 (9)$^{**}$ | 39 (10)$^{*}$ | 39 (6)$^{***}$ | 46 (12)$^{*}$ | 34 (7)$^{**}$ | 61 (17)$^{*/\alpha\alpha}$ |
| **Glucose (mg/dL)** | 109 (5) | 120 (10) | 120 (9) | 104 (6) | 142 (14)$^{\beta\beta}$ | 108 (9) | 164 (15)$^{*/\alpha\alpha\alpha/\beta\beta}$ | 110 (10) | 179 (14)$^{**/\alpha\alpha\alpha/\beta\beta}$ |
| **Nutritional parameters** | | | | | | | | | |
| - Cholesterol (mg/dL) | 223 (11) | 170 (13)$^{**}$ | 183 (8)$^{**}$ | 179 (6)$^{*}$ | 170 (12)$^{*}$ | 181 (8)$^{**}$ | 175 (12)$^{*}$ | 183 (8)$^{**}$ | 165 (9)$^{*}$ |
| - Triglycerides (mg/dL) | 119 (12) | 143 (31) | 98 (8) | 112 (12)$^{*}$ | 100 (14) | 93 (7) | 149 (28) | 112 (12)$^{*}$ | 120 (20) |
| - Proteins (g/dL) | 7.2 (0.1) | 7.3 (0.2) | 7.4 (0.1) | 7.4 (0.1) | 7.3 (0.2) | 7.4 (0.1) | 7.4 (0.1) | 7.0 (0.4) | 7.4 (0.2) |
| **Kidney function** | | | | | | | | | |
| - Urea (mg/dL) | 38 (3) | 37 (4) | 35 (2) | 33 (3) | 41 (4) | 34 (4) | 47 (8) | 29 (3)$^{*}$ | 38 (7) |
| - Creatinine (mg/dL) | 0.8 (0.05) | 0.8 (0.05) | 0.8 (0.04) | 0.8 (0.04) | 0.9 (0.09) | 0.8 (0.06) | 0.9 (0.15) | 0.8 (0.05) | 1.1 (0.2) |
| - Sodium (mEq/L) | 138 (0.3) | 139 (0.8) | 138 (0.8) | 138 (0.9) | 138 (2) | 139 (0.8) | 139 (1.3) | 139 (1.1) | 138 (1.5) |
| **Liver test** | | | | | | | | | |
| - AST (U/L) | 25 (1) | 50 (6)$^{***}$ | 40 (4)$^{***}$ | 43 (5)$^{**}$ | 34 (5) | 48 (5)$^{***}$ | 34 (3)$^{*}$ | 45 (6)$^{**}$ | 39 (5)$^{*}$ |
| - ALT (U/L) | 24 (2) | 34 (3)$^{**}$ | 31 (2)$^{*}$ | 33 (3)$^{*}$ | 28 (3) | 34 (3)$^{**}$ | 26 (3) | 33 (4) | 27 (2) |
| - GGT (U/L) | 32 (7) | 109 (20)$^{**}$ | 80 (10)$^{***}$ | 81 (10)$^{***}$ | 78 (21) | 76 (12)$^{**}$ | 76 (16)$^{*}$ | 71 (9)$^{**}$ | 89 (22)$^{*}$ |
| - ALP (mU/mL) | 76 (9) | 125 (12)$^{**}$ | 132 (11)$^{***}$ | 142 (15)$^{***}$ | 115 (18) | 148 (13)$^{***}$ | 100 (14) | 132 (15)$^{*}$ | 108 (24) |
| **Liver function** | | | | | | | | | |
| - Bilirubin (mg/dL) | 0.6 (0.02) | 1.3 (0.2)$^{**}$ | 1.7 (0.4)$^{**}$ | 1.9 (0.6)$^{*}$ | 1.3 (0.3)$^{*}$ | 1.9 (0.6) | 1.4 (0.3)$^{*}$ | 1.5 (0.4) | 1.5 (0.4)$^{*}$ |
| - Albumin (g/dL) | 4.6 (0.07) | 3.8 (0.1)$^{***}$ | 3.7 (0.1)$^{***}$ | 3.7 (0.1)$^{***}$ | 3.8 (0.2)$^{***}$ | 3.7 (0.1)$^{***}$ | 3.7 (0.2)$^{*}$ | 3.7 (0.1)$^{***}$ | 3.7 (0.3)$^{*}$ |
| - INR | 1.0 (0.0) | 1.2 (0.1)$^{**}$ | 1.2 (0.0)$^{***}$ | 1.2 (0.1)$^{***}$ | 1.1 (0.1)$^{*}$ | 1.2 (0.05)$^{***}$ | 1.2 (0.07)$^{*}$ | 1.3 (0.07)$^{***}$ | 1.2 (0.05)$^{*}$ |
| **Blood count** | | | | | | | | | |
| - Leucocytes (x$10^9$/L) | 6.80 (0.7) | 5.69 (0.4) | 5.4 (0.4) | 4.8 (0.3) | 5.3 (0.5) | 4.8 (0.4)$^{*}$ | 4.6 (0.5)$^{*}$ | 5.4 (0.5) | 5.7 (0.6) |
| - Haemoglobin (g/dL) | 14.6 (0.2) | 13.4 (0.4)$^{*}$ | 13.4 (0.3)$^{**}$ | 13.2 (0.4)$^{*}$ | 13.0 (0.6) | 13.5 (0.5) | 12.5 (0.7)$^{*}$ | 13.9 (0.5)$^{***}$ | 12.8 (0.6)$^{**}$ |
| - Platelets (x$10^9$/L) | 241 (15) | 116 (11)$^{***}$ | 119 (11)$^{***}$ | 105 (9)$^{***}$ | 107 (11)$^{***}$ | 105 (12)$^{***}$ | 107 (17)$^{***}$ | 114 (13) | 122 (15)$^{***}$ |

**Supplementary Table S1.** Biochemical parameters according to rifaximin response. All values are expressed as mean (SEM). *significant differences from controls (*$p$<0.05; **$p$<0.01; ***$p$<0.001). $^{\alpha}$ significant differences from no MHE ($^{\alpha}p$<0.05; $^{\alpha\alpha}p$<0.01; $^{\alpha\alpha\alpha}p$<0.001). $^{\beta}$significant differences from responders ($^{\beta}p$<0.05; $^{\beta\beta}p$<0.01; $^{\beta\beta\beta}p$<0.001).$^{\partial}$significant differences from baseline ($^{\partial}p$<0.05; $^{\partial\partial}p$<0.01; $^{\partial\partial\partial}p$<0.001). Abbreviations: AST: aspartate aminotransferase; ALT: alanine aminotransferase; GGT: gamma glutamyl transferase; ALP: Alkaline phosphatase; INR: international normalized ratio. MHE and No MHE: patients with or without minimal hepatic encephalopathy, respectively.

| Psychometric test | | Patients (n=63) | | Baseline | | 3-month follow-up | | 6-month follow-up | |
|---|---|---|---|---|---|---|---|---|---|
| | Controls (n=30) | No MHE (n=31) | MHE (n=32) | Responders (n=21) | Non responders (n=11) | Responders (n=21) | Non responders (n=11) | Responders (n=19) | Non responders (n=9) |
| **COGNITIVE TEST** | | | | | | | | | |
| **Stroop test** | | | | | | | | | |
| - Congruent task | 115 (2.6) | 105 (3.2)$^{*}$ | 71 (4)$^{***/\alpha\alpha\alpha}$ | 73 (5)$^{***/\alpha\alpha\alpha}$ | 67 (7)$^{***/\alpha\alpha\alpha}$ | 80 (5)$^{***/\alpha\alpha\alpha}$ | 67 (5)$^{***/\alpha\alpha\alpha}$ | 85 (5)$^{***/\alpha\alpha/\partial}$ | 64 (9)$^{***/\alpha\alpha\alpha/\beta}$ |
| - Neutral task | 83 (2.4) | 75 (2.3)$^{*}$ | 56 (3)$^{***/\alpha\alpha\alpha}$ | 58 (3)$^{***/\alpha\alpha\alpha}$ | 51 (3)$^{***/\alpha\alpha\alpha}$ | 63 (3)$^{***/\alpha\alpha}$ | 52 (3)$^{***/\alpha\alpha\alpha/\beta}$ | 64 (3)$^{***/\alpha\alpha/\partial}$ | 52 (4)$^{***/\alpha\alpha}$ |
| - Incongruent task | 46 (1.5) | 41 (2)$^{*}$ | 29 (2)$^{***/\alpha\alpha\alpha}$ | 31 (3)$^{***/\alpha\alpha}$ | 25 (2)$^{***/\alpha\alpha\alpha}$ | 35 (2)$^{***/\alpha}$ | 27 (3)$^{***/\alpha\alpha\alpha/\beta}$ | 35 (2)$^{***/\alpha/\partial}$ | 34 (8)$^{*}$ |
| **d2 test** | | | | | | | | | |
| - Total responses | 402 (16) | 337 (13)$^{**}$ | 244 (17)$^{***/\alpha\alpha\alpha}$ | 269 (19)$^{***/\alpha\alpha}$ | 188 (26)$^{***/\alpha\alpha\alpha/\beta}$ | 307 (18)$^{***}$ | 179 (18)$^{***/\alpha\alpha\alpha/\beta\beta}$ | 295 (19)$^{***}$ | 219 (40)$^{**/\alpha}$ |
| - Total correct | 150 (6.4) | 130 (6)$^{*}$ | 83 (7)$^{***/\alpha\alpha\alpha}$ | 93 (8)$^{***/\alpha\alpha\alpha}$ | 60 (13)$^{***/\alpha\alpha\alpha/\beta}$ | 111 (9)$^{***}$ | 64 (10)$^{***/\alpha\alpha\alpha/\beta\beta}$ | 108 (9)$^{***/\alpha}$ | 71 (19)$^{***/\alpha\alpha/\partial\partial}$ |
| - Omission errors | 17 (2.6) | 12 (2) | 21 (4) | 22 (6) | 19 (6) | 20 (4) | 11 (3) | 16 (6) | 22 (2) |
| - Commission errors | 1 (0.2) | 4 (1)$^{*}$ | 11 (3)$^{**/\alpha}$ | 10 (4)$^{*}$ | 12 (5) | 3 (1)$^{\partial}$ | 6 (4) | 6 (3) | 14 (6)$^{\alpha\alpha}$ |
| - Total effectiveness | 371 (19) | 298 (16)$^{**}$ | 214 (16)$^{***/\alpha\alpha}$ | 237 (18)$^{***/\alpha}$ | 161 (29)$^{***/\alpha\alpha\alpha/\beta}$ | 284 (19)$^{**/\partial}$ | 161 (21)$^{***/\alpha\alpha\alpha/\beta\beta}$ | 274 (19)$^{**}$ | 184 (47)$^{**/\alpha}$ |
| - Concentration index | 146 (6.7) | 126 (6)$^{*}$ | 72 (9)$^{***/\alpha\alpha\alpha}$ | 83 (10)$^{***/\alpha\alpha\alpha}$ | 49 (14)$^{***/\alpha\alpha\alpha}$ | 108 (19)$^{**/\partial}$ | 58 (12)$^{***/\alpha\alpha\alpha/\beta\beta}$ | 102 (11)$^{***/\alpha}$ | 57 (25)$^{***/\alpha\alpha}$ |
| **Oral SDMT** | 50 (1.3) | 44 (2)$^{**}$ | 23 (2)$^{***/\alpha\alpha\alpha}$ | 29 (2)$^{***/\alpha\alpha\alpha}$ | 18 (3)$^{***/\alpha\alpha\alpha/\beta}$ | 33 (2)$^{***/\alpha\alpha\alpha}$ | 20 (2)$^{***/\alpha\alpha\alpha/\beta\beta}$ | 37 (2)$^{***/\alpha}$ | 20 (4)$^{***/\alpha\alpha\alpha/\beta\beta}$ |
| **Digit Span** | 16 (0.8) | 13 (0.5)$^{**}$ | 10 (0.5)$^{***/\alpha\alpha}$ | 11 (0.7)$^{***/\alpha}$ | 9 (0.7)$^{***/\alpha\alpha\alpha}$ | 11 (0.9)$^{***}$ | 9 (0.7)$^{***/\alpha\alpha}$ | 13 (2)$^{*}$ | 10 (2)$^{**}$ |
| **Letter-Number sequencing** | 10 (0.4) | 8 (0.5)$^{**}$ | 5 (0.5)$^{***/\alpha\alpha\alpha}$ | 6 (0.9)$^{***/\alpha\alpha}$ | 4 (0.8)$^{***/\alpha\alpha\alpha}$ | 7 (0.9)$^{***}$ | 5 (0.8)$^{***/\alpha\alpha}$ | 7 (1)$^{***}$ | 3 (1)$^{***/\alpha\alpha\alpha}$ |
| **MOTOR COORDINATION TESTS** | | | | | | | | | |
| **Bimanual coordination** | 1.9 (0.03) | 2.3 (0.1)$^{***}$ | 3.7 (0.4)$^{***/\alpha\alpha}$ | 3.0 (0.2)$^{***/\alpha\alpha\alpha}$ | 4.7 (0.8)$^{**/\alpha}$ | 2.6 (0.1)$^{***/\alpha\alpha/\partial\partial}$ | 3.7 (0.3)$^{***/\alpha\alpha\alpha/\beta\beta}$ | 2.7 (0.1)$^{***/\alpha\alpha}$ | 4.0 (0.5)$^{*/\alpha/\beta}$ |
| **Visuo-motor coordination** | 2.4 (0.1) | 3 (0.1)$^{***}$ | 3.9 (0.2)$^{***/\alpha\alpha\alpha}$ | 3.5 (0.2)$^{***/\alpha\alpha}$ | 4.5 (0.3)$^{***/\alpha\alpha\alpha/\beta\beta}$ | 3.4 (0.2)$^{***/\alpha}$ | 4.8 (0.7)$^{**/\alpha}$ | 3.3 (0.3)$^{**}$ | 4.7 (0.4)$^{**/\alpha\alpha\alpha/\beta}$ |

**Supplementary Table S2.** Psychometric test performance at baseline and according to rifaximin response. All values are expressed as mean (SEM). *significant differences from controls (*$p$<0.05; **$p$<0.01; ***$p$<0.001). $^{\alpha}$ significant differences from no MHE ($^{\alpha}p$<0.05; $^{\alpha\alpha}p$<0.01; $^{\alpha\alpha\alpha}p$<0.001). $^{\beta}$significant differences from responders ($^{\beta}p$<0.05; $^{\beta\beta}p$<0.01; $^{\beta\beta\beta}p$<0.001). $^{\partial}$significant differences from baseline ($^{\partial}p$<0.05; $^{\partial\partial}p$<0.01; $^{\partial\partial\partial}p$<0.001). Abbreviations: MHE and No MHE: patients with or without minimal hepatic encephalopathy, respectively.

| Biochemical parameters | Controls (n=30) | Patients (n=63) | | Baseline | | 3-month follow-up | | 6-month follow-up | |
|---|---|---|---|---|---|---|---|---|---|
| | | No MHE (n=31) | MHE (n=32) | Responders (n=21) | Non responders (n=11) | Responders (n=21) | Non responders (n=11) | Responders (n=19) | Non responders (n=9) |
| **IMMUNOPHENOTYPE STUDY** | | | | | | | | | |
| **Monocytes**[a] | | | | | | | | | |
| - Classical | 92.2 (0.7) | 88.7 (0.8)$^{**}$ | 89.7 (0.9)$^{*}$ | 89.6 (1.2) | 90 (1.5) | 92 (0.8)$^{/\alpha}$ | 89.7 (1.3) | 93 (0.6)$^{\alpha\alpha\alpha/\partial}$ | 88.6 (2.1) |
| - Intermediate | 3.3 (0.3) | 7.5 (0.6)$^{***}$ | 9.4 (0.8)$^{***/\alpha}$ | 8.7 (0.7)$^{***}$ | 11.1 (2.2)$^{*}$ | 6.1 (0.6)$^{***/\partial}$ | 7.5 (1.3)$^{*/\partial}$ | 5.3 (0.5)$^{***/\alpha/\partial\partial}$ | 10.4 (2.1)$^{*}$ |
| - Non-classical | 2 (0.3) | 2.1 (0.3) | 2.7 (0.4) | 3 (0.4) | 1.9 (1.0) | 1.2 (0.3)$^{\alpha/\partial}$ | 0.5 (0.2)$^{***/\alpha}$ | 0.6 (0.2)$^{***/\alpha\alpha\alpha/\partial\partial\partial}$ | 0.5 (0.4)$^{*/\alpha\alpha}$ |
| **$CD4^+$T lymphocytes** | | | | | | | | | |
| - Autoreactive[b] | 8 (1) | 10 (1) | 12 (2)$^{*}$ | 10.8 (2) | 15 (4) | 14 (4) | 14 (3) | 7 (2) | 12 (4) |
| - Non-autoreactive[b] | 92 (3) | 90 (1) | 88 (2) | 90 (2) | 85 (4) | 85 (5) | 87 (3) | 92 (2) | 89 (5) |
| - Activated[c] | 0.5 (0.1) | 1 (0.2)$^{**}$ | 2 (0.4)$^{**/\alpha}$ | 3 (1)$^{**/\alpha}$ | 0.8 (0.2)$^{*/\beta\beta}$ | 1.2 (02)$^{**}$ | 0.9 (0.1)$^{**}$ | 1 (0.2)$^{*}$ | 0.9 (0.2)$^{*}$ |
| **CYTOKINES**[d] | | | | | | | | | |
| IL-6 | 0.9 (0.1) | 2.0 (0.2)$^{***}$ | 2.2 (0.3)$^{***}$ | 2.2 (0.4)$^{**}$ | 2.2 (0.6)$^{*}$ | 1.8 (0.4)$^{*}$ | 1.5 (0.6) | 1.2 (0.3)$^{\alpha}$ | 1.0 (0.5) |
| IL-18 | 152 (12) | 207 (22)$^{*}$ | 229 (25)$^{**}$ | 253 (36)$^{*}$ | 184 (17) | 228 (28)$^{*}$ | 153 (41) | 234 (36)$^{*}$ | 146 (43) |
| IL-15 | 2.9 (0.1) | 3.3 (0.1)$^{*}$ | 3.4 (0.1)$^{*}$ | 3.5 (0.6)$^{**}$ | 3.1 (0.7) | 3.2 (0.1) | 3.1 (0.2) | 2.9 (0.1)$^{\alpha/\partial\partial}$ | 3 (0.2) |
| IL-17 | 1.3 (0.1) | 2.3 (0.4)$^{*}$ | 2.7 (0.4)$^{**}$ | 2.7 (0.5)$^{**}$ | 2.6 (0.5)$^{*}$ | 2.7 (0.5)$^{*}$ | 3.2 (1.0) | 1.9 (0.3) | 4.5 (1)$^{\beta\beta}$ |
| IL-21 | 155 (14) | 237 (33)$^{*}$ | 439 (143) | 503 (211) | 312 (87) | 320 (163) | 438 (119) | 294 (206) | 638 (130)$^{*/\alpha\alpha\alpha}$ |
| IL-22 | 57 (2.5) | 63 (2.6) | 99 (6.8)$^{***/\alpha\alpha\alpha}$ | 103 (6.7)$^{***/\alpha\alpha\alpha}$ | 93 (16) | 89 (6.8)$^{***/\alpha\alpha/\partial\partial}$ | 95 (10)$^{*}$ | 84 (11)$^{*/\partial\partial}$ | 98 (16)$^{*}$ |
| CXCL13 | 59 (2.0) | 115 (6.7)$^{***}$ | 155 (13)$^{***/\alpha\alpha}$ | 165 (18)$^{***/\alpha\alpha}$ | 138 (19)$^{**}$ | 156 (20)$^{***/\alpha}$ | 174 (39)$^{*}$ | 122 (26)$^{\partial}$ | 203 (58) |
| CX3CL1 | 0.6 (0.03) | 0.7 (0.04)$^{**}$ | 0.9 (0.1)$^{***/\alpha\alpha}$ | 0.9 (0.1)$^{***/\alpha\alpha}$ | 1.0 (0.1)$^{*}$ | 0.9 (0.1)$^{***/\alpha}$ | 1.0 (0.1)$^{***/\alpha\alpha}$ | 0.9 (0.1)$^{**}$ | 1.1 (0.1)$^{*/\alpha}$ |
| CCL20 | 8.9 (0.9) | 64 (8.8)$^{***}$ | 79 (11)$^{***}$ | 88 (13)$^{***}$ | 56 (21) | 59 (10)$^{***/\partial}$ | 47 (9)$^{**}$ | 45 (8)$^{***/\partial\partial}$ | 46 (6)$^{**}$ |
| **TRANSCRIPTION FACTORS**[e] | | | | | | | | | |
| BCL6 | 1.0 (0.1) | 0.8 (0.1)$^{*}$ | 0.9 (0.1) | 0.9 (0.1) | 1.0 (0.3) | 0.9 (0.1) | 0.7 (0.2) | 1.0 (0.1) | 1.2 (0.2) |
| AHR | 0.9 (0.1) | 1.0 (0.1) | 1.4 (0.2)$^{**/\alpha}$ | 1.5 (0.2)$^{*/\alpha}$ | 1.2 (0.3) | 1.3 (0.2)$^{*}$ | 1.2 (0.2)$^{*}$ | 1.0 (0.1)$^{\partial}$ | 0.9 (0.1) |
| TBX21 | 1.1 (0.1) | 1.4 (0.1)$^{*}$ | 1.4 (0.1)$^{*}$ | 1.3 (0.1) | 1.7 (0.3) | 1.5 (0.1)$^{*}$ | 1.4 (0.1) | 1.2 (0.1) | 1.5 (0.4) |
| GATA3 | 1.1 (0.1) | 1.0 (0.1) | 1.0 (0.1) | 0.9 (0.1) | 1.1 (0.1) | 1.2 (0.2)$^{\partial}$ | 1.0 (0.1) | 1.2 (0.1) | 0.9 (0.2) |
| RORC | 1.0 (0.1) | 0.9 (0.1) | 0.8 (0.1) | 0.8 (0.1) | 0.8 (0.1) | 0.7 (0.1)$^{*}$ | 0.6 (0.1)$^{**}$ | 0.5 (0.1)$^{***/\alpha\alpha}$ | 0.4 (0.1)$^{**}$ |
| **IgG levels**[f] | 99 (2) | 89 (20) | 155 (12)$^{**}$ | 153 (3) | 155 (2) | 124 (9)$^{\partial}$ | 160 (7)$^{\beta}$ | 101 (6)$^{\partial\partial}$ | 169 (21)$^{\beta}$ |

**Supplementary Table S3.** Inflammatory parameters at baseline and according to rifaximin response. [a]Expressed as percentage of the three subsets of monocytes over total monocyte cells. [b]Expressed as percentage of total $CD4^+$T lymphocytes. [c]Percentage of $CD4^+$ T lymphocytes that express the early activation marker CD69. [d]Cytokine levels are expressed in pg/mL except for CX3CL1 which is in ng/mL. [e]Data represent the normalized target gene (HPRT) amount relative to controls which are considered as 1. [f]Percentage of variation compared to controls. All values are expressed as mean (SEM). *significant differences from controls (*$p$<0.05; **$p$<0.01; ***$p$<0.001). $^{\alpha}$significant differences from no MHE ($^{\alpha}p$<0.05;$^{\alpha\alpha}p$<0.01; $^{\alpha\alpha\alpha}p$<0.001). $^{\beta}$Significant differences from responders ($^{\beta}p$<0.05; $^{\beta\beta}p$<0.01; $^{\beta\beta\beta}p$<0.001). $^{\partial}$significant differences from baseline ($^{\partial}p$<0.05; $^{\partial\partial}p$<0.01; $^{\partial\partial\partial}p$<0.001). Abbreviations: MHE and No MHE: patients with or without minimal hepatic encephalopathy, respectively.
